# Optogenetic Control of Gene Expression in *Drosophila*

**DOI:** 10.1371/journal.pone.0138181

**Published:** 2015-09-18

**Authors:** Yick-Bun Chan, Olga V. Alekseyenko, Edward A. Kravitz

**Affiliations:** Department of Neurobiology, Harvard Medical School, Boston, Massachusetts, United States of America; University of Houston, UNITED STATES

## Abstract

To study the molecular mechanism of complex biological systems, it is important to be able to artificially manipulate gene expression in desired target sites with high precision. Based on the light dependent binding of cryptochrome 2 and a cryptochrome interacting bHLH protein, we developed a split lexA transcriptional activation system for use in *Drosophila* that allows regulation of gene expression *in vivo* using blue light or two-photon excitation. We show that this system offers high spatiotemporal resolution by inducing gene expression in tissues at various developmental stages. In combination with two-photon excitation, gene expression can be manipulated at precise sites in embryos, potentially offering an important tool with which to examine developmental processes.

## Introduction

Cellular and developmental processes require precise temporal and positional control of the patterns of activation and expression of genes. To help unravel the molecular mechanism of complex biological systems, it is valuable for researchers to artificially manipulate gene expression with high precision. The classical approaches for doing this use cell type specific or chemically inducible promoters to drive gene expression: these provide considerable maneuverability, but usually have poor temporal resolution and limited spatial specificity. More recently, several promising optogenetic approaches have been developed that focus on light sensitive proteins. These offer unmatched advantages in manipulating gene expression *in vivo* since light is non invasive and can be directed with exceptionally high spatiotemporal resolution.

A variety of light-inducible systems have been developed based on photoactivatable adenylyl cyclase [1], transduction cascade components [2], and caged small-molecule inducers [3]. However, the possibility of off-target effects and the requirements for specific chemical inducers with these systems have limited their usability. A more direct approach for regulating gene expression is through coupling a light dependent protein-protein interaction pair in a yeast two-hybrid-like assay system. Each protein partner is linked to either a DNA binding or a transcriptional activation domain of a specific transcription factor in order to build a functional factor with light induction. Based on the known red light induced binding of phytochrome and the phytochrome interacting factor (PIF), several red/far-red light switchable expression systems of this type already have been developed [4,5]. These provide the advantage of rapid stimulation and reversibility.

More recently, studies have focused on other light sensitive protein interactions such as the binding of FKF1 and GIGANTEA proteins [6,7], the interaction of the UVR8 and COP1 proteins [8], and homo-dimerization of the ‘light-oxygen-voltage’ (LOV) domain [9,10]. Among molecular partners of this type, the blue light dependent binding of cryptochrome 2 (CRY2) to its partner cryptochrome interacting protein (CIB), has been the most studied as this system offers rapid on- and off-kinetics of protein pair-binding within minutes and requires no added co-factors. A light switchable transcription system of this type has yielded promising results *in vitro* and *in vivo* in mouse and zebrafish studies [11–13]. The ability to induce this system using two-photon excitation allows, in principle, for fine spatial manipulation of gene expression *in vivo*. To fully realize the potential of this technology, we developed a light inducible transcription system for use in *Drosophila melanogaster* by combining the split LexA system [14] with the light-activated cryptochrome from *Arabidopsis*. Exposure to blue light leads to the formation of functional LexA transcription factor and subsequently turns on gene expression downstream of LexAOP promoter. We optimized the system using S2 cell culture and demonstrated over 10-fold gene up-regulation after brief exposure to blue light. Next, we validated the system *in vivo* using embryos and adults. Using a LexAOP2-GFP reporter line, we observed significant GFP expression after just 2 hours of blue light exposure. We also demonstrated the adaptability and functionality of this system using GAL4 line to target different neural populations with GCaMP3.0 reporter and functional effectors like TrpA1. This system provides a novel approach to regulate gene expression and is especially suitable for neurodevelopmental studies that require precise spatial and temporal regulation.

## Materials and Methods

### Fly stocks

Fly stocks were maintained at 25°C on standard medium. All crosses used for light induction experiments were kept in darkness all the time. Collection of flies was performed in a dark room under dim red light. The following stocks were used in this study: LexAOP2-mCD8::GFP (#32205) [15], LexAOP2-Gal80 (#32213) [15], *orco*-Gal4 (#23292) [16], GMR18G07-Gal4 (#45448) [17], *elav* ^*c155*^-Gal4 (#458) (# from Bloomington Stock Center), *fru*-Gal4 [18], LexAOP-TrpA1 [19], LexAOP-GCaMP3.0 (gift from Dr. Orie Shafer). The UAS-CIBN::LexA-mcherry-p65::CRY2 line was generated by a PhiC31 mediated, site-specific insertion of a pUAS-CIBN::LexA-mcherry-p65::CRY2 construct into an attP2 site [20] (Genetic Services, Inc).

### DNA constructs

#### pActPL-CIBN-LexA

The leucine zipper of pActPL-LexADBD [14] was removed by a *Bam*HI-*Nhe*I cut and replaced with the N-terminus of CIB (CIBN, amino acids 1–171), that was generated by PCR amplification from pmCherry-CIBN-CreC (Addgene #26889).

#### pActPL-vp16-CRY2

The VP16AD and its poly-glycine linker was obtained from a pActPL-VP16AD [14] by a *Bam*HI-*Spe*I digestion and inserted into pPacPL (gift from Dr. Thomas Schwarz) to generate the pPacPL-vp16-linker construct. The full-length CRY2 was PCR amplified from pmcherry-CRY2-CreN (Addgene #26888) and cloned (*Xba*I) into pPacPL-vp16-linker plasmid to obtain a pActPL-vp16-CRY2 construct.

#### pActPL-p65-CRY2

The VP16AD sequence was removed (*Bam*HI-*Nhe*I) from a pPacPL-vp16-linker construct and replaced with a transcription activation domain of p65 (amino acids 281–548) that was amplified from a pBPLexA::p65Uw construct (Addgene #26231). Then the full-length CRY2 was inserted into an *Xba*I restriction site to generate the pActPL-p65-CRY2 construct.

#### pUAS-CIBN::LexA-mcherry-p65::CRY2

T2A sequences were incorporated into both forward and reverse primers to amplify the full-length mcherry from pmcherry-CRY2-CreN. The amplified mcherry sequence was inserted into a *Bgl*II-*Xba*I digested pJFRC177 vector (Addgene #32149). CIBN::LexA and p65::CRY2 were amplified from pActPL-CIBN-LexA and pActPL-p65-CRY2, and cloned into *Bgl*II and *Xba*I restriction sites respectively using the Gibson Assembly method. The resulting UAS-CIBN::LexA-mcherry-p65::CRY2 construct was confirmed by sequencing before proceeding with the generation of the transgenic line (S1 Fig).

### Cell culture and light induction

S2 cells were cultured in standard Schneider’s medium (Life Technology). For each of the DNA constructs, equal amounts of plasmid (1μg) were added for transfection using an Effectene Transfection Reagent (QIAGEN) with a DNA:Effectene ratio of 1:12.5. After two days of incubation in the dark at room temperature, the transfected cells were re-suspended and plated onto individual coverslips in culture dishes. Dishes were either illuminated with a blue LED (474nm ~2.5mWcm^-2^) or were kept in the dark for 1 hour. Equivalent numbers of cells were plated on each coverslip to allow direct light-dark comparisons. After that, experimental (illuminated) and control culture dishes were incubated in the dark for a further 4 hours to allow time for transcription and translation, fixed with 4% formaldehyde, and subsequently washed three times with PBS before mounting for observation. Ten fields per coverslip were captured and analyzed automatically using ImageJ software to calculate the average numbers of GFP positive cells. The following parameters were used in the ImageJ analysis: “Otsu threshold”, “Fill holes”, “Watershed”, and “Circularity 0.4–1.0” to identify GFP positive cells.

### Light illumination

#### Embryo illumination

Embryos were collected onto 1% agarose plates and incubated at 25°C in the dark for 12 hours. They then were illuminated from above using a blue LED (474nm) at intensity levels and exposure times reported in the figures (S2 Fig). After light induction, embryos were dechorionated in 50% bleach for ~5min and fixed with 4% formaldehyde according to a standard protocol [21]. For induction using two photon microscopy, embryos were dechorionated, aligned and glued onto coverslips as prepared for embryo microinjection. Hydrocarbon oil was used to cover the embryos to prevent them from desiccation during two photon induction (S2 Fig). Embryos were induced using a Zeiss LSM510 microscope equipped with a Coherent Chameleon^TM^ Multi-photon excitation laser (860nm, 80MHz, ~200fs pulse width, 5% power) for 4 hours.

#### Adult illumination

CO_2_ anesthetized adult flies were transferred to a thin transparent tube (TriKinetics, PPT5x65) to ensure even exposure to light. Tubes were then illuminated from above using a blue LED (474nm) with intensities and exposure times as specified in the figures. Immediately after light induction, brains were dissected and fixed in 4% formaldehyde for 20–30 min before proceeding to immunostaining as described next.

### Immunohistochemistry

Fixed tissues were washed with PBT (1x PBS, 0.1% Triton-X) at room temperature 3 times for 15–20 min per wash. Then they were washed in a blocking solution (PBT, 0.5% BSA) for ~1 hour before incubating with mouse anti-GFP antibodies (Invitogen, 1:500) overnight at 4°C. Following 3 more rounds of washes with PBT and one wash with the blocking solution as before, tissues were incubated with Alexa Fluor 488 anti-mouse IgG (Invitrogen, 1:300) for ~4 hours at room temperature. This was followed by 3 rounds of washes with PBT and then samples were mounted with Vectashield. Fluorescence images were acquired using a Nikon eclipse 90i fluorescence microscope. Structured illumination microscopy (Optigrid) was used to obtain optical sections (1.8μm thickness) of the entire central brain region. Image J software was used to generate Z-stack projections and to measure GFP intensity.

### Measurement of GFP intensity

Z-stack projections of the embryonic nervous system was obtained using structured illumination microscopy (Optigrid) and ImageJ software. Average GFP intensity of the nervous system in embryos was measured and normalized to the average background intensity outside the nervous system. For measurement of GFP intensity levels in adult brains, due to large variations in the intensity levels between cells near the surface and cells deeper within the brain, total GFP intensity was scored instead of measuring the average GFP intensity. Total GFP intensity was calculated by summing the GFP signal in each pixel after subtraction of a background signal. Images were acquired using same microscopy setting and exposure time without post-modification in each experiment to ensure correct quantification.

### Quantitative polymerase chain reaction

Transgenic males (*elav* ^*c155*^-Gal4 UAS-CIBN::LexA-mcherry-p65::CRY2 LexAOP2-GFP) with or without the LexAOP2-Gal80 feedback mechanism were either exposed to blue light (474nm, ~2.5mWcm^-2^) for ~18 hours or kept in constant darkness. Flies were frozen in liquid nitrogen immediately after the light induction and fly heads were collected from 10–15 flies for each sample. Total RNA was extracted using TRIzol (Life Technologies) following the manufacturer’s protocol. cDNA was generated using the QuantiTect Reverse Transcription Kit (QIAGEN). Quantitative polymerase chain reaction (qPCR) on GFP was performed using iQ^TM^ SYBR Green Supermix and CRX96 System (Bio-Rad). Three technical replicas were included in each sample and the results were normalized against the mRNA level of actin to determine the **Δ**Ct (cycle threshold) values. Relative fold change in mRNA amount was calculated using the **Δ**Ct value of the un-induced flies (*elav*-Gal4 UAS-CIBN::LexA-mcherry-p65::CRY2 LexAOP2-GFP LexAOP2-Gal80) as the reference.

### Live imaging of GCaMP3.0 response

Transgenic flies expressing GCaMP3.0 in *fruitless* neurons (*fru*-Gal4 UAS-CIBN::LexA-mcherry-p65::CRY2 LexAOP-GCaMP3.0) were either exposed to blue light (474nm, ~2.5mWcm^-2^) for ~24 hours or kept in constant darkness. Flies were anesthetized on ice and the brains were dissected directly into the hemolymph-like (HL3) buffer containing 70mM NaCl, 5mM KCl, 1.5mM CaCl_2_, 20mM MgCl_2_, 10mM NaHCO_3_, 5mM trehalose, 115mM sucrose, and 5mM HEPES (pH 7.1). All cuticle, compound eye and large trachea were removed from the dissected brain. Brains were mounted anterior surface up in the drop of HL3 buffer placed in the center of 35-mm Falcon dish. A petri dish insert for a PS-8H perfusion system (Bioscience Tools, San Diego, CA) was lowered around the brain and the brain was allowed to recover for 10 min. HL3 flow was established across the brain in the beginning of each experiment with PS-8H gravity-fed perfusion system (Bioscience Tools, San Diego, CA). The cholinergic agonist carbamylcholine chloride (10^−3^ M Carbachol, Sigma-Aldrich, St. Louis, MO) was applied by switching the flow from the main HL3 buffer line to the second line containing the test compound for 60 sec, followed by return to the HL3 flow line. The working concentration of bath-applied carbachol and the detailed description of the method were reported previously [22]. We used the same experimental conditions for both light-induced brains and for the control brains. Each brain was stimulated with carbachol only once. Live imaging of GCaMP3.0 sensor was conducted on the Nikon Eclipse 90i microscope equipped with a Photometrics Coolsnap K4 and CFI Fluor 20X N.A. 0.5, W.D. 2mm dipping cone objective. The brains were first located based on the basal expression of the sensor using green fluorescent protein optics. Images were taken using the time lapse setting with 1 sec intervals for 5 min. The same exposure time (40 ms) was used for both the experimental and control groups. A small region of interest around the mcAL *fru*-positive cluster was selected for quantification of changes in fluorescent intensity. Mean pixel intensities of GCaMP3.0 fluorescence was calculated for each time point.

### Behavioral assay

Transgenic flies expressing the light–inducible transcription system and TrpA1 in *fruitless* neurons (*fru*-Gal4 UAS-CIBN::LexA-mcherry-p65::CRY2 LexAOP-TrpA1) were incubated at 19°C. Males were collected and exposed to blue light (474nm, ~2.5mWcm^-2^) in thin transparent tubes (TriKinetics, PPT5x65) for ~24 hours or kept in continuous darkness. For the behavioral assay, flies were aspirated to a 12-well plate (Falcon) containing ~2 ml fly food and videotaped for 30 min inside a 30°C incubator.

## Results

### Generation of a light inducible transcription system

**Fig 1:**
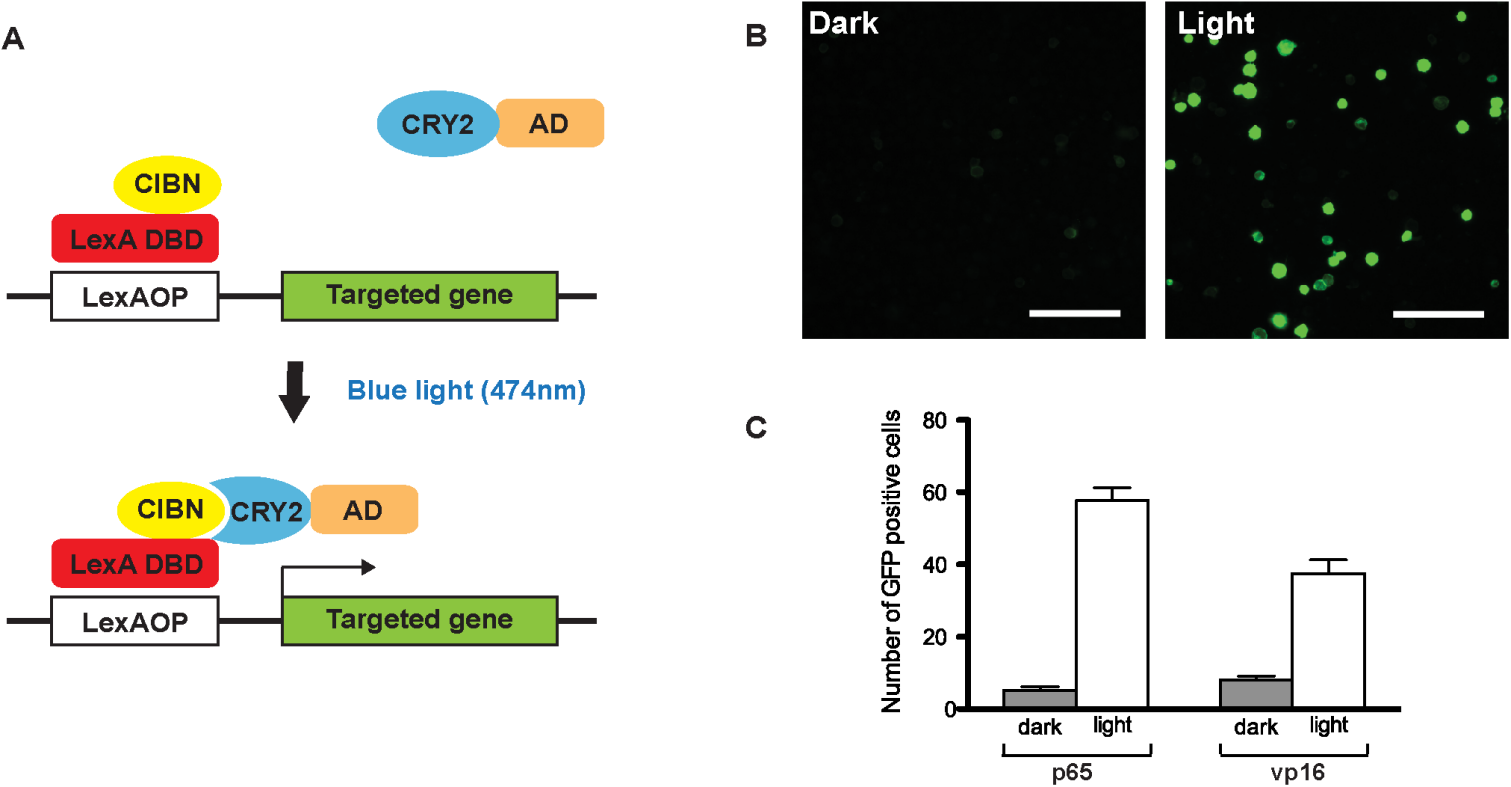
A light inducible transcription system. (A) Schematic representation of the light inducible LexA transcription system. Upon blue light (474nm, ~2.5mWcm^-2^) illumination, CRY2 undergoes a conformational change that allows it to bind to CIBN to form a functional LexA transcription activator. (B) Light induced GFP expression in S2 cells. S2 cells were transfected with the two constructs that should generate a functional transcription factor upon blue light illumination (pActPL-CIBN-LexA and pActPL-p65-CRY2) and a LexAOP-GFP reporter construct. Cells were exposed to blue light for 1 hour or kept in the dark. (Scale bar = 100μm) (C) Comparison of the efficacy of the VP16 and p65 transcription activation domains. The average number of GFP positive cells per field of view (n = 10, mean±s.e.m. t-test p<0.0001).

To generate a light inducible transcription system, we first fused a LexA DNA binding domain to the N-terminus of the CIB protein (CIBN, amino acid 1–171), and a transcriptional activator to the CRY2 protein. (Fig 1A). For these experiments, we used the full length CRY2 protein instead of the reportedly more efficient N-terminal photolyase homology region (CRY2PHR) because the baseline activity of the intact protein in the dark is lower [12]. We felt this to be an important consideration in attempting to generate a clean ON-OFF switch for a targeted gene expression system [12]. To examine the efficiency of this light induction system in a *Drosophila* test tissue, we co-transfected S2 cells with both constructs and with a LexAOP-GFP reporter. We also examined the efficiency of the activation domains of two different transcription factors: one from the herpes viral protein VP16 and the other from the human transcription factor p65. Significant GFP expression was observed in both cases after one-hour of continuous exposure to blue light (474nm, ~2.5mWcm^-2^) (Fig 1B). We evaluated the efficacy of the different activation domains by counting the numbers of GFP positive cells. A greater fold change in cell number and a lower basal activity in the dark was observed with the p65 activation domain (Fig 1C). Therefore, this activation domain was used in our CRY2 constructs in all subsequent experiments.

### Optogenetic control of gene expression *in vivo*

**Fig 2:**
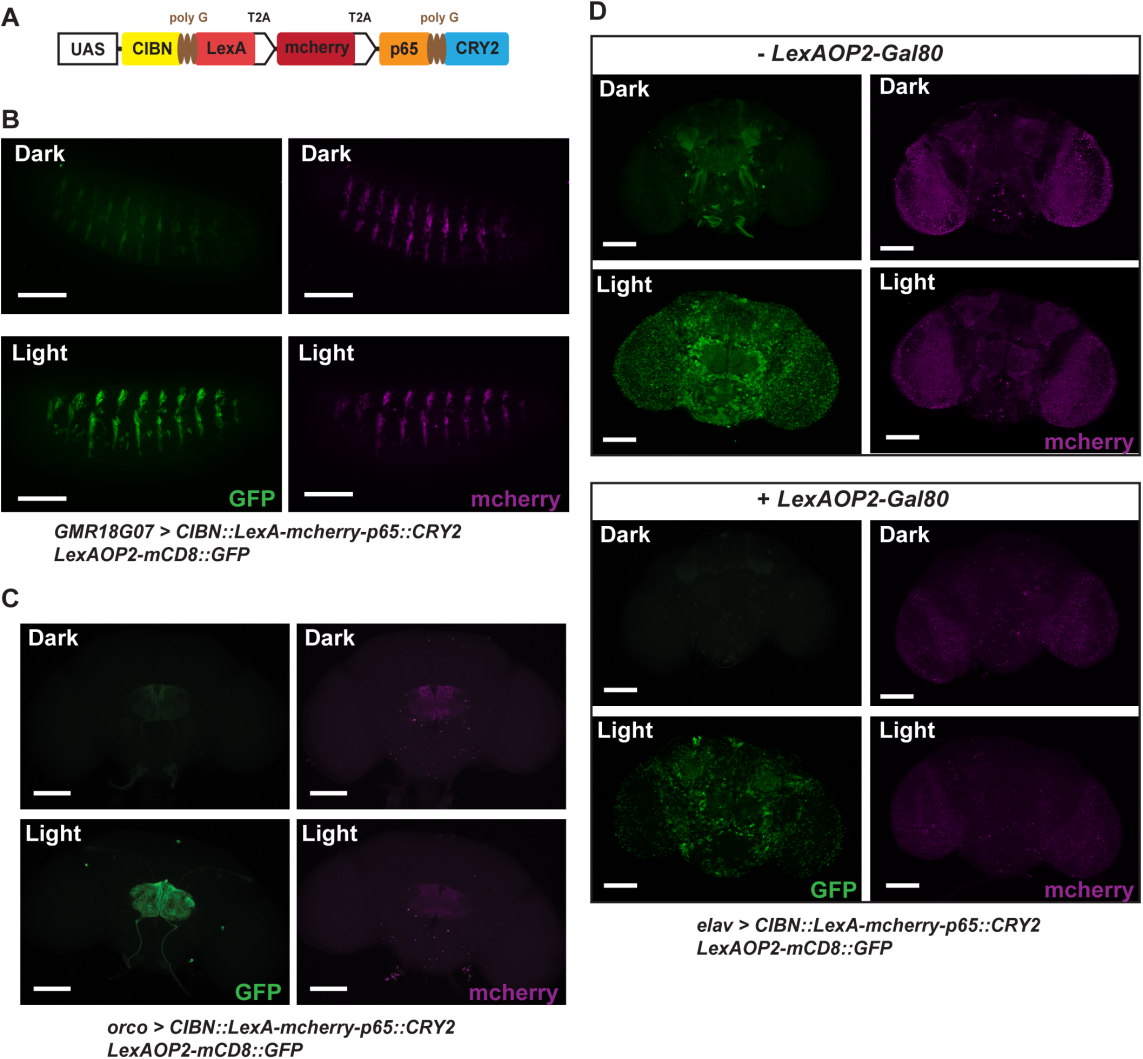
*In vivo* use of the light-inducible gene expression system. (A) Schematic representation of the transgenic construct carrying the total light inducible LexA transcription system. T2A ribosomal skipping signals were added to the C-terminus of CIBN::LexA and mcherry to maintain stoichiometry of the required proteins. (B, C) Induction of GFP expression using the light switchable system in embryos (B) and adults (C). GMR18G07-Gal4 and orco-Gal4 were used to drive the transcription system in embryos and in adults respectively. GFP expression was seen in neuroblast cells in embryos or antennal lobe neurons in adults after blue light illumination (embryo: 4 hours; 474nm, ~1.1mWcm^-2^; adult ~16 hours, 474nm, ~2.5mWcm^-2^). (D) Addition of a Gal80 feedback mechanism minimizes basal expression without illumination. *elav* ^*c155*^-Gal4 were used to drive the transcription system in adults. Flies were illuminated with blue light for ~16 hours or kept in constant darkness. The incorporation of LexAOP2-Gal80 dramatically lowered the basal GFP expression in control without blue light illumination. This construct also greatly reduced the expression of the transcription system marked by mcherry. The reduction in mcherry expression was observed in samples with the LexAOP2-Gal80 feedback mechanism even if the exposure time was 6 times longer than that of samples without the LexAOP2-Gal80 (Scale bar = 100μm).

As next steps in building a transcriptional system that can be targeted to different tissues in intact flies, we put the constructs involved with the entire CRY2/CIBN light induction system under the control of a UAS promoter region (Fig 2A). To minimize the number of transgenes involved and to maintain the stoichiometry of expression of CRY2::p65 and CIBN::LexA, we added a T2A ribosomal skipping signal to the C-terminus of the CIBN::LexA fusion protein [23]. We also incorporated an mcherry marker with a second T2A signal at its C-terminus between the CRY2 and CIBN constructs, to visualize the cells for light induction. With this larger construct, all three proteins should be generated separately under control of the same promoter. We tested the efficacy of this system in embryos with a GMR18G07-Gal4 driver that targets subset of neuroblasts in each segment in stage 15 embryos. After 4 hours of continuous blue light exposure (474nm, ~1.1mWcm^-2^), we observed clear GFP expression in the induced sample when compared to the negative control that had been left in the dark (Fig 2B and S3 Fig). To see whether this system can be used in freely moving adults with intact cuticles, we expressed the light inducible transcription system in olfactory receptor neurons using an *orco*-Gal4 driver. Flies were placed in narrow tubes and exposed to blue light continuously for ~16 hours, after which brains were dissected and imaged. The results showed highly significant GFP expression in the antennal lobes (Fig 2C and S3 Fig). In these experiments, we expect that gene expression levels can vary depending on the length of exposure, the intensity of light, and the stability of the target proteins and mRNAs. We observed substantial GFP expression as early as 1 hour of light exposure in embryos or 2 hours in adults.

Another factor that can influence expression levels is the strength of the Gal4 drivers used. To explore this, we tested several strong neuronal Gal4 driver lines like *elav* ^*c155*^-Gal4. With the stronger drivers, we observed very large increases in GFP expression after blue light induction. However, we also observed increased basal levels of expression of GFP even in non-induced controls. Previous studies have reported that the CRY2-CIBN system is somewhat leaky and displays some basal transcriptional activity [12,13]. Therefore, it may not be surprising that in our system a strong Gal4 driver would lead to higher background levels of gene expression. To eliminate or reduce this undesired effect of driver strength, we added a negative feedback mechanism to our system by incorporating a LexAOP2-Gal80 transgene. Any basal transcription of our CRY2-CIBN construct in the absence of light induction also will lead to the expression of Gal80, which in turn will down regulate the function of Gal4 and lower the strength of the driver. Using this approach, we successfully reduced the level of background expression when using strong Gal4 drivers (Fig 2D). The background reduction was confirmed by measuring GFP-mRNA levels and the entire system was usable with a variety of different Gal4 drivers (S4 Fig). No lethality was seen in adults even after 24 hours of light induction (n>10) and no significant effect on survival rate was observed in embryos (S3 Fig). This suggests that the duration and intensity of the light induction did not affect survival. As anticipated, the effect was wavelength specific as we saw little GFP expression with red light stimulation (S5 Fig).

### Spatiotemporal control of gene expression using light

**Fig 3:**
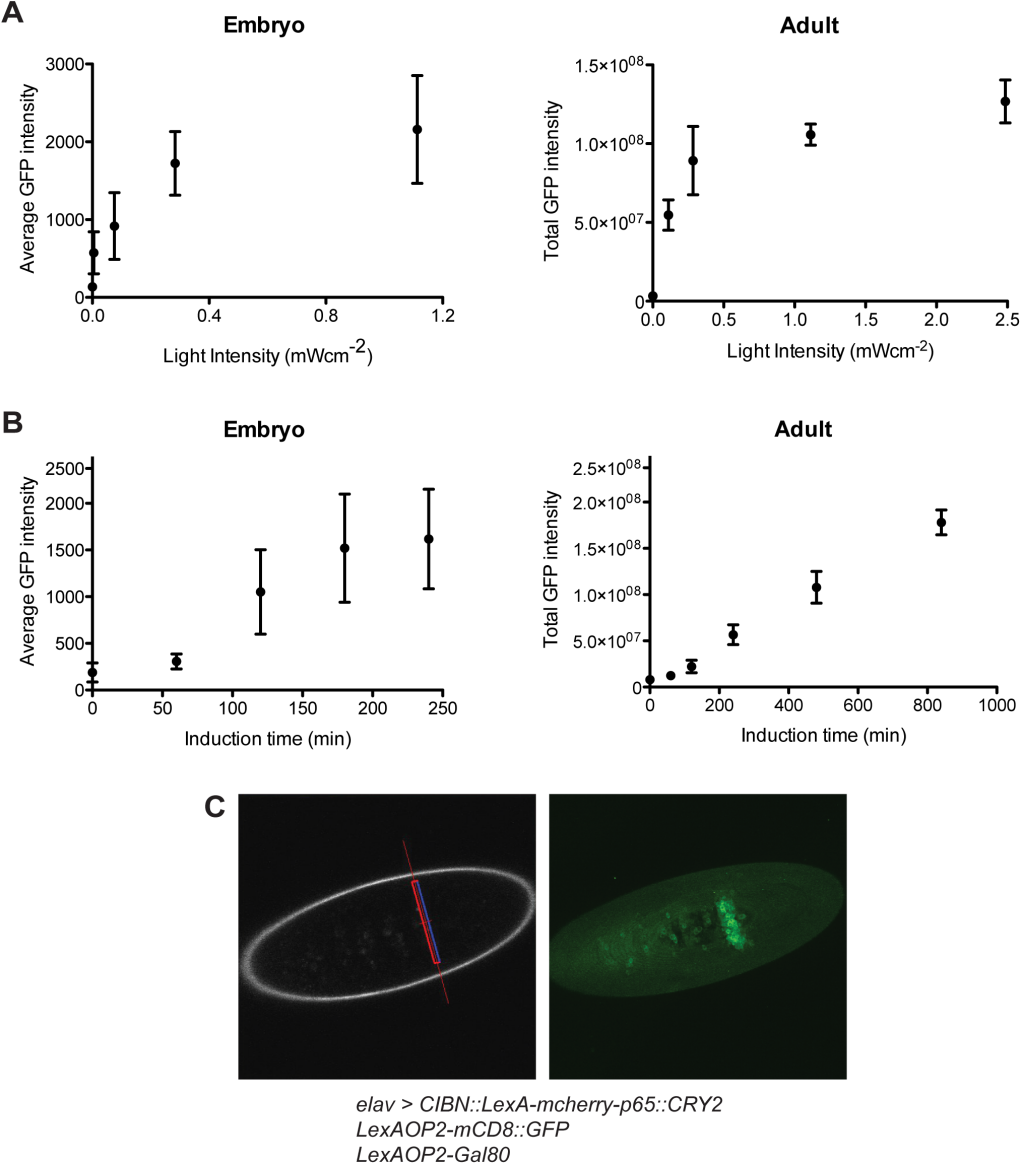
Kinetics of the light inducible transcription system driven by *elav* ^*c155*^-Gal4 with LexAOP2-Gal80 feedback control. (A) Effect of light intensity on GFP expression levels in embryos and adults. The average GFP intensity of the nervous system was measured in embryos after 4 hours of blue light illumination (n>10, mean±s.d.). The total GFP intensity of the whole brain was measured in the adults because of an uneven GFP distribution among the various brain regions. Adult flies were illuminated with continuous blue light for ~16 hours (n>5, mean±s.d.). (B) Effect of exposure time on GFP expression levels in embryos and adults. The GFP expression levels in the nervous system were measured after illuminating embryos or adults with blue light (474nm, ~2.5mWcm^-2^) for the specified periods of time (n>7, mean±s.d.). (C) Selective spatial induction of gene expression using two-photon excitation. The region of interest is highlighted with the red line (left image) and exposed to two photon excitation (860nm, 80MHz, 200fs pulse width) for 4 hours. The Z-stack projection of the GFP expression pattern in the nervous system was imaged immediately afterwards (right image). A 3D-reconstruction of the exposed region is shown in S1 Movie (Scale bar = 100μm).

Most other available genetic tool systems in *Drosophila* such as the Gal4/UAS system offer reliable spatial resolution but provide limited control of gene expression level. To evaluate the potential of our system in this regard, we used the complete light inducible transcription system including Gal80 feedback to examine the effects of light intensity, the temporal kinetics of induction, and the spatial resolution of the system. *Light-Induction thresholds*: Since light intensity can be adjusted in our system, fine-tuning of the levels of gene expression should be possible (Fig 3A). Indeed, GFP expression in live animals can be seen at light intensities as low as 16μWcm^-2^ in embryos and ~100μWcm^-2^ in adults. The higher light intensity required in adults is likely due to the opacity of the cuticle. *Temporal control of induction*: Gaining tight and rapid control of gene expression has been challenging in *Drosophila*. For example, expression systems using temperature-sensitive Gal80 or drug-induced gene-switch elements show response times of over several hours [24,25]. More rapid responses are seen using a heat shock promoter, but this system commonly lacks ease of spatial regulation [26]. To explore the speed of gene expression in our system, we examined the effects of exposure time under standardized illumination conditions (474nm, ~2.5mWcm^-2^) in embryos and adult flies. In embryos with the Gal80 feedback mechanism, significant expression of GFP was observed after 2 hours of exposure, an effect that reached a plateau after 3 hours (Fig 3B). Although 2–3 hours response time is relatively longer than the fast response from a heat shock promoter, this system allows some spatial control depending on the Gal4 drivers used. In adults, with their darker cuticles, GFP expression was found after ~4 hours of light exposure that increased gradually over time. This temporal response was comparable to or better than other currently used expression systems. *Spatial resolution*: A special advantage of using light as the trigger for gene induction is the potential for excellent spatial resolution. To explore this potential, we first tested the use of a confocal microscope to focus on targets of interest. We quickly found, however, that the high sensitivity of our system led to strong basal expression in surrounding non-targeted tissues as well. To minimize this spread, we used a two-photon microscope instead. In comparison with blue light excitation, the far-red laser used in two-photon excitation shows less scattering and deeper penetration of the photon beam into tissues. We demonstrated that focusing the two-photon beam to a narrow band (~10μm) in embryos resulted in distinct selective GFP expression in the targeted region (Fig 3C and accompanying S1 Movie). The region displaying GFP expression (~20μm) is wider than the initially exposed target area. This could be a combined result of light scattering, slight movements of embryos and cell migration from the exposed area during embryonic development. However, even though a strong pan-neuronal driver (i.e. *elav* ^*c155*^-Gal4) was used in these experiments, little basal expression was observed in the surrounding non-induced, more distant tissues. Such good spatial and temporal resolution could be of particular value for developmental studies.

### Optogenetic control of GCaMP3.0 expression in *fruitless* neurons

**Fig 4:**
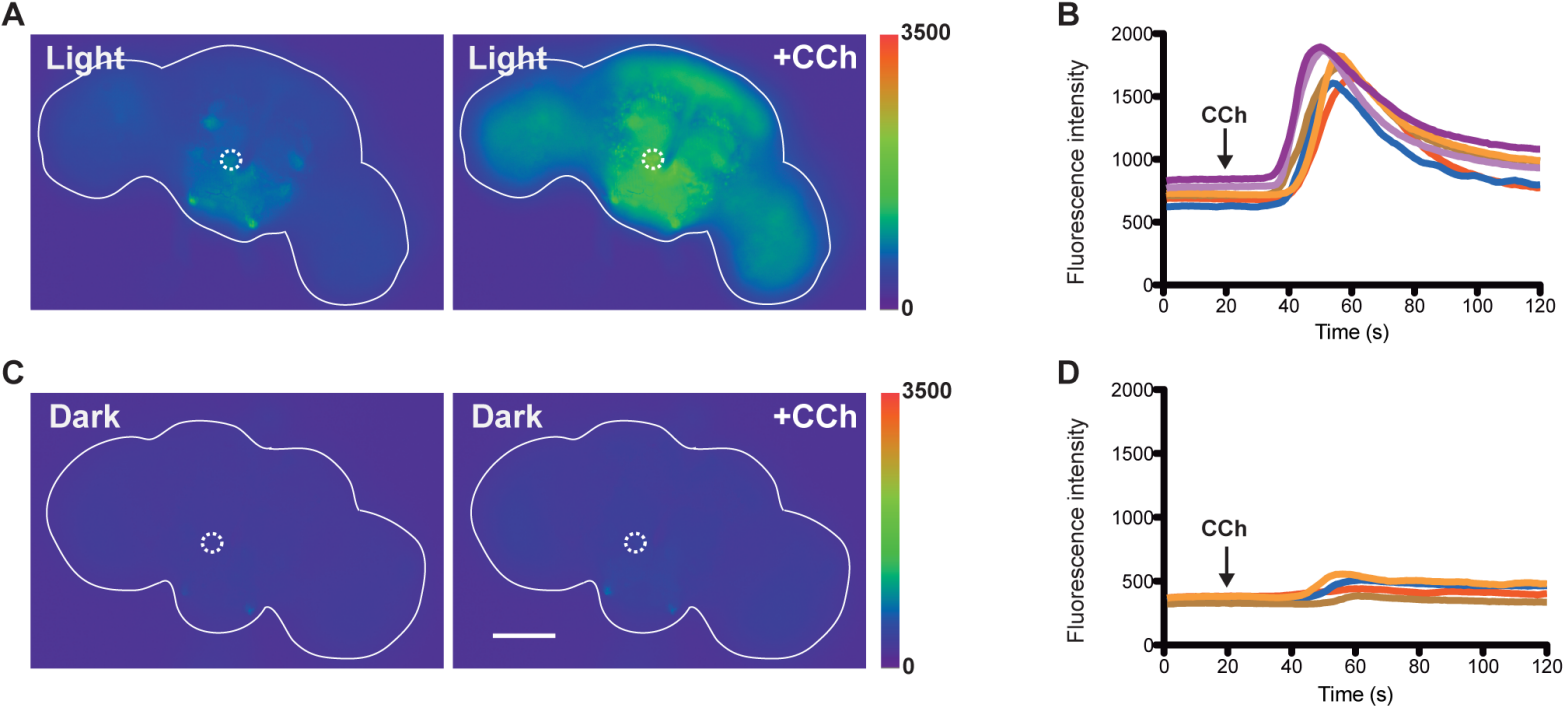
Live imaging of GCaMP3.0 fluorescence in *fruitless* neurons. (A) Males expressing the light–inducible LexA system (*fru*-Gal4 UAS-CIBN::LexA-mcherry-p65::CRY2 LexAOP-GCaMP3.0) were exposed to blue light (474nm, ~2.5mWcm^-2^) for 24 hours. Clear increase in GCaMP3.0 fluorescence was observed after the administration of 10mM carbamylcholine. (B) Time-lapse changes in GCaMP3.0 fluorescence in the mcAL neuronal cluster region (highlighted in dotted circle). Each line represented a response curve from an individual brain (n = 6). The arrow indicates when the perfusion of carbamylcholine (CCh) started (~20 sec). (C) Males expressing the light–inducible LexA system were kept in darkness before the assay. Little changes in GCaMP3.0 fluorescence was observed after the administration of 10mM carbamylcholine. (D) Time-lapse changes in GCaMP3.0 fluorescence in the mcAL cluster (highlighted in dotted circle). Each line represents a response curve from an individual brain (n = 4).

To illustrate the adaptability of the light inducible transcription system for other transgenes, we used the system to express calcium sensors in specific neurons as monitors of neuronal function. In *Drosophila*, sexually dimorphic courtship behaviors are regulated by about 1500 fruitless (Fru^M^)-expressing neurons in the nervous system [18]. To visualize neural activity in these neurons we used our light-inducible transcription system to selectively express a calcium sensor, GCaMP3.0, in the Fru^M^-population of neurons, through targeting these neurons with a *fru*-Gal4 driver. Only in animals that were pre-exposed to blue light should GCaMP3.0 have been synthesized in Fru^M^-positive neurons and showed changes in fluorescence upon activation [27]. Exposure of adult males to blue light (474nm, ~2.5mWcm^-2^) did lead to the selective expression of the GCaMP3.0 sensor in these neurons. Basal GCaMP3.0 fluorescent signals are observed in Fru^M^-positive neural clusters (e.g. mcAL—dotted circle on figures) in males exposed to blue light (Fig 4A), but not in the control kept in constant darkness (Fig 4C). To induce neural activities, we perfused the dissected fly brains with the cholinergic agonist carbamylcholine (CCh). Large increases in GCaMP3.0 fluorescence were observed only in brains with prior exposure to blue light (Fig 4A and 4B, and accompanying S2 Movie). Several brain regions including the protocerebrum, mushroom bodies, antennal lobes and optic lobes also showed clear increases of fluorescence. Little changes in GCaMP3.0 fluorescence was observed in the control without blue light exposure (Fig 4C and 4D). This experiment demonstrates the applicability of the light-inducible transcription system for the regulation of expression of a calcium sensor in a neural circuit that allows imaging of real-time neural activity.

### Optogenetic control of courtship behavior

**Fig 5:**
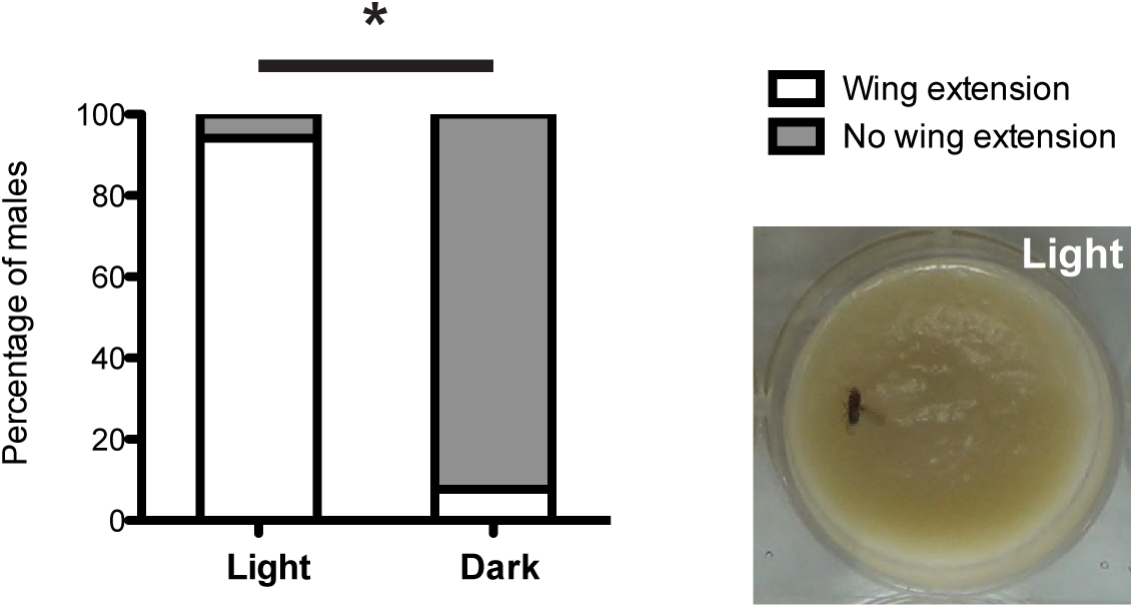
Display of courtship behavior in males expressing TrpA1 in *fruitless* neurons using the light-inducible transcription system. Males expressing the light-inducible LexA system (*fru*-Gal4 UAS-CIBN::LexA-mcherry-p65::CRY2 LexAOP-TrpA1) displayed significantly more unilateral wing extensions at 30°C, a typical courtship behavioral pattern, after blue light exposure (474nm, ~2.5mWcm^-2^) for 24 hours (p<0.001, Fisher’s exact test, n = 17 and 13 for light and dark respectively). The photo shows a typical wing extension behavior displayed by males with prior exposure to blue light.

Next, we moved on to explore the utility of this system for the manipulation of behavior in *Drosophila*. In this case, with blue light pre-exposure of flies, we drove synthesis of the heat-sensitive cation channel TrpA1 instead of a calcium sensor in Fru^M^-expressing neurons [28]. Previous studies had shown that activation of the *fruitless* circuit by TrpA1 leads to robust displays of patterns associated with courtship behavior in males, including unilateral wing extensions and abdominal bending [29]. In males without exposure to blue light, we observed essentially no courtship behavior during the 30 min period of 30°C incubation that was needed to activate the TrpA1 channel (Fig 5). Only in a single case, a brief wing extension (~1 sec) was observed. In contrast, many observations of typical courtship behavioral patterns, like abdominal bending and unilateral wing extensions, were seen during the 30°C incubation of males with prior exposure to blue light (Fig 5 and accompanying S3 and S4 Movies). These results demonstrate that the light-inducible transcription system functions well to control TrpA1 expression and in that way can be used to regulate the expression of behavioral patterns *in vivo*.

## Discussion

A great strength of *Drosophila* research is the existence of excellent genetic tools that allow us to study gene function with good spatial and temporal resolution. However, there are still many limitations in the current techniques. For instance, combinatorial approaches using the Gal4/UAS technique with the FLP/FRT method or the Gal80 system allow some degree of spatial regulation, but large screens are usually required to identify useful lines [30]. In terms of temporal control, the temperature sensitive Gal80 and gene-switch approaches provide some long-term regulation, but the induction times are long [24,25]. Using a heat-shock promoter allows tight temporal control, but lacks spatial restriction. In order to develop a new approach that can achieve both spatial and temporal control in a single system, we have turned to optical induction and have developed a new light inducible transcription system for use in *Drosophila*. Recently, Boulina et al. reported the use of cryptochrome 2 and Cre/LoxP recombination systems for cell imaging [31]. Here we have further developed the system to achieve both spatial and temporal control of gene expression *in vivo* and have demonstrated its applicability in inducing gene expression in embryonic and adult tissues. This may be particularly valuable for developmental studies during embryonic life as embryos are transparent. In adults, this system can compliment currently available genetic tools in facilitating our understanding of complex biological systems like neural circuits that require fine spatiotemporal resolution.

There are, however, some limitations intrinsic to the current system. One major potential problem is the basal expression in uninduced samples. For certain Gal4 lines like *orco*-Gal4, we observed only weak levels of basal expression. However, with other driver lines like *elav* ^*c155*^, significant levels of basal expression were seen. These results indicated that background expression levels varied from one driver to another. To mitigate the effects of strength differences in Gal4 lines, we introduced a Gal80 feedback mechanism. This can successfully reduce the level of basal gene expression. However, the introduction of additional transgenes can complicate the genetic crosses and might lead to unforeseen effects with certain phenotypes. At present, it is possible to incorporate the feedback mechanism into the same transgene cassette. In the long run, with further optimization of protein sequences within the construct, it should be possible to enhance the sensitivity and reduce the signal-to-noise ratio even further. Ultimately, this will allow us to completely eliminate the need for the Gal80 feedback mechanism.

Even in its current form, however, the ability to control gene expression levels and the high spatiotemporal resolution available with this system, offer unique advantages for the study of gene function in fruit flies. By the use of two photon excitation, we demonstrated restricted spatial induction in the range of ~20μm. In theory, this level of resolution could allow us to target specific neural clusters in central brain regions and manipulate gene expression during development. Depending on the Gal4 driver used, further optimization of the induction conditions should allow us to regulate gene expression with even higher spatial resolution.

In addition to driving expression of GFP to test our system, however, we also demonstrated several potential uses of the light-inducible transcription system to study neural function by driving the expression of the calcium sensor GCaMP3.0 and the cation channel TrpA1 in Fru^M^-producing neurons. With the calcium sensor experiments, application of the cholinergic agonist carbamyl choline led to dramatic increases in GCaMP3.0 fluorescence only in flies that had been pre-exposed to blue light. No changes were observed in control flies that had been kept in continuous darkness. These results are encouraging and we anticipate that in combination with two photon light induction and further optimization of the technique, it will be possible to restrict light induction to only smaller subsets of neurons. Hence, this procedure potentially offers a novel way for investigators to express key molecular entities useful for *in vitro* and *in vivo* manipulation of neurons, circuits and ultimately, behavior.

The light-inducible transcription system described here demonstrates proof of concept and several potential uses for a novel approach towards the manipulation of gene expression in *Drosophila*. This method compliments other genetic tools and has the potential to provide high spatiotemporal resolution that may be of benefit in developmental, neurobiological and behavioral studies.

## Supporting Information

S1 Fig Complete sequence of the *CIBN*::*LexA-mcherry-p65*::*CRY2* transgene. (DOCX)

S2 Fig Setup for light illumination. (A) Embryos were collected onto 1% agarose plates and illuminated from above using a commercial blue LED (474nm) (Superbrightleds MR16). (B) Schematic representation of the setup for two photon induction of embryo. Dechorionated embryos were aligned and glued onto coverslips. The coverslips were placed on top of a hole in a 35mm petri dish (MatTek). Wet filter paper and hydrocarbon oil was introduced to prevent embryos from desiccation during two photon induction.
(TIF)

S3 Fig Quantification of GFP expression. (A) Quantification of GFP expression in neuroblast cells in embryos. GMR18G07-Gal4 was used to drive the UAS- light inducible transcription system in neuroblast cells. Stage 15 embryos were illuminated with blue light (474nm, ~1.1mWcm^-2^) for 4 hours. Significant GFP expression was observed after light induction (n = 20, mean±s.e.m., t-test p<0.0001). (B) Quantification of GFP expression in olfactory neuron in adult flies after blue light induction. *orco*-Gal4 was used to drive the UAS- light inducible transcription system selectively in olfactory neurons. Adult flies were exposed to blue light (474nm, ~2.5mWcm^-2^) for 24 hours. Significant GFP expression was observed in the antennal lobes after light induction (n = 10, mean±s.e.m., t-test p<0.0001). (C) No significant effects were seen on survival rates of embryos. Embryos expressing the light inducible transcription system in the nervous system using a pan neuronal driver (*elav* ^*c155*^-Gal4 UAS-CIBN::LexA-mcherry-p65::CRY2 LexAOP2-GFP LexAOP2-Gal80) were illuminated with blue light (474nm, ~2.5mWcm^-2^) throughout the entire period of embryogenesis. No lethality was observed comparing to the negative control incubated in dark (Fisher’s exact test, p>0.4).
(TIF)

S4 Fig Reduced background expression using the LexAOP2-Gal80 feedback mechanism. (A) Quantification of the GFP mRNA expression level using qPCR. Transgenic male flies (*elav* ^*c155*^-Gal4 UAS-CIBN::LexA-mcherry-p65::CRY2 LexAOP2-GFP) with or without the LexAOP2-Gal80 feedback mechanism were either exposed to blue light (474nm, ~2.5mWcm^-2^) for ~18 hours or kept in constant darkness. Relative fold changes in mRNA amount were normalized against the un-induced flies with the LexAOP2-Gal80 feedback mechanism (n = 3, mean±s.e.m.). (B) Addition of a Gal80 feedback mechanism minimizes basal expression without illumination in *fruitless* neurons. Flies were illuminated with blue light for ~16 hours or kept in constant darkness. The incorporation of LexAOP2-Gal80 successfully lowered the basal GFP mRNA expression (Scale bar = 100μm).
(TIF)

S5 Fig Effect of light color on the light inducible transcription system. Induction of gene expression using the light switchable system depends on the wavelength of light. *elav* ^*c155*^-Gal4 was used, including the LexAOP2-Gal80 feedback system, to drive the light inducible transcription system in adults. Flies were exposed to either red (626nm) or blue light (474nm, ~2.5mWcm^-2^) for 16 hours. Clear GFP expression was observed in adults illuminated with blue light only. Exposure to red light yielded little induction of GFP expression (Scale bar = 100μm).
(TIF)

S1 Movie 3D-reconstruction of ventral nervous system in embryo. Regions exposed to two photon excitation showed clear GFP expression.
(MP4)

S2 Movie Time-lapse video of GCaMP3.0 fluorescent response. Time-lapse video of Fru^M^-expressing neurons demonstrates GCaMP3.0 fluorescence before and after the administration of the cholinergic agonist carbamoylcholine. The video was played at 30 frames per second representing a total of 120 sec in real time.
(MP4)

S3 Movie Display of unilateral wing extension in male flies at 30°C. Exposure to blue light (474nm, ~2.5mWcm^-2^) for 24 hours led to the expression of LexAOP-TrpA1 in Fru^M^-expressing neurons. Activation of TrpA1 at 30°C resulted in displays of courtship behavior (e.g. wing extension) in the absence of female flies.
(MP4)

S4 Movie Display of abdominal bending in male flies at 30°C. Exposure to blue light (474nm, ~2.5mWcm^-2^) for 24 hours led to the expression of LexAOP-TrpA1 in Fru^M^-expressing neurons. Activation of TrpA1 at 30°C resulted in displays of courtship behavior (e.g. abdominal bending) in the absence of female flies.
(MP4)
