# Supplementary material for: Optogenetic Control of Gene Expression in Drosophila
Source: PLoS One. 2015 Sep 18;10(9):e0138181. doi: 10.1371/journal.pone.0138181 (PMC4575133; doi:10.1371/journal.pone.0138181)
Supplement: S1 Fig — (DOCX) [file pone.0138181.s001.docx]

Figure 1. Complete sequence of the *CIBN::LexA-mcherry-p65::CRY2* transgene construct.

pUAS-**CIBN-LexA-T2A-**mcherry-T2A**-p65-CRY2**

GACTCAGGTGGCACTTTTCGGGGAAATGTGCGCGGAACCCCTATTTGTTTATTTTTCTAAATACATTCAA

ATATGTATCCGCTCATGAGACAATAACCCTGATAAATGCTTCAATAATATTGAAAAAGGAAGAGTATGAG

TATTCAACATTTCCGTGTCGCCCTTATTCCCTTTTTTGCGGCATTTTGCCTTCCTGTTTTTGCTCACCCA

GAAACGCTGGTGAAAGTAAAAGATGCTGAAGATCAGTTGGGTGCACGAGTGGGTTACATCGAACTGGATC

TCAACAGCGGTAAGATCCTTGAGAGTTTTCGCCCCGAAGAACGTTTTCCAATGATGAGCACTTTTAAAGT

TCTGCTATGTGGCGCGGTATTATCCCGTATTGACGCCGGGCAAGAGCAACTCGGTCGCCGCATACACTAT

TCTCAGAATGACTTGGTTGAGTACTCACCAGTCACAGAAAAGCATCTTACGGATGGCATGACAGTAAGAG

AATTATGCAGTGCTGCCATAACCATGAGTGATAACACTGCGGCCAACTTACTTCTGACAACGATCGGAGG

ACCGAAGGAGCTAACCGCTTTTTTGCACAACATGGGGGATCATGTAACTCGCCTTGATCGTTGGGAACCG

GAGCTGAATGAAGCCATACCAAACGACGAGCGTGACACCACGATGCCTGTAGCAATGGCAACAACGTTGC

GCAAACTATTAACTGGCGAACTACTTACTCTAGCTTCCCGGCAACAATTAATAGACTGGATGGAGGCGGA

TAAAGTTGCAGGACCACTTCTGCGCTCGGCCCTTCCGGCTGGCTGGTTTATTGCTGATAAATCTGGAGCC

GGTGAGCGTGGGTCTCGCGGTATCATTGCAGCACTGGGGCCAGATGGTAAGCCCTCCCGTATCGTAGTTA

TCTACACGACGGGGAGTCAGGCAACTATGGATGAACGAAATAGACAGATCGCTGAGATAGGTGCCTCACT

GATTAAGCATTGGTAACTGTCAGACCAAGTTTACTCATATATACTTTAGATTGATTTAAAACTTCATTTT

TAATTTAAAAGGATCTAGGTGAAGATCCTTTTTGATAATCTCATGACCAAAATCCCTTAACGTGAGTTTT

CGTTCCACTGAGCGTCAGACCCCGTAGAAAAGATCAAAGGATCTTCTTGAGATCCTTTTTTTCTGCGCGT

AATCTGCTGCTTGCAAACAAAAAAACCACCGCTACCAGCGGTGGTTTGTTTGCCGGATCAAGAGCTACCA

ACTCTTTTTCCGAAGGTAACTGGCTTCAGCAGAGCGCAGATACCAAATACTGTTCTTCTAGTGTAGCCGT

AGTTAGGCCACCACTTCAAGAACTCTGTAGCACCGCCTACATACCTCGCTCTGCTAATCCTGTTACCAGT

GGCTGCTGCCAGTGGCGATAAGTCGTGTCTTACCGGGTTGGACTCAAGACGATAGTTACCGGATAAGGCG

CAGCGGTCGGGCTGAACGGGGGGTTCGTGCACACAGCCCAGCTTGGAGCGAACGACCTACACCGAACTGA

GATACCTACAGCGTGAGCTATGAGAAAGCGCCACGCTTCCCGAAGGGAGAAAGGCGGACAGGTATCCGGT

AAGCGGCAGGGTCGGAACAGGAGAGCGCACGAGGGAGCTTCCAGGGGGAAACGCCTGGTATCTTTATAGT

CCTGTCGGGTTTCGCCACCTCTGACTTGAGCGTCGATTTTTGTGATGCTCGTCAGGGGGGCGGAGCCTAT

GGAAAAACGCCAGCAACGCGGCCTTTTTACGGTTCCTGGCCTTTTGCTGGCCTTTTGCTCACATGTTACC

GTCGACGATGTAGGTCACGGTCTCGAAGCCGCGGTGCGGGTGCCAGGGCGTGCCCTTGGGCTCCCCGGGC

GCGTACTCCACCTCACCCATCTGGTCCATCATGATGAACGGGTCGAGGTGGCGGTAGTTGATCCCGGCGA

ACGCGCGGCGCACCGGGAAGCCCTCGCCCTCGAAACCGCTGGGCGCGGTGGTCACGGTGAGCACGGGACG

TGCGACGGCGTCGGCGGGTGCGGATACGCGGGGCAGCGTCAGCGGGTTCTCGACGGTCACGGCGGGCATG

TCGACAAGCCGAACATATGGGCGCGCCTAGTATGTATGTAAGTTAATAAAACCCATTTTTGCGGAAAGTA

GATAAAAAAAACATTTTTTTTTTTTACTGCACTGGATATCATTGAACTTATCTGATCAGTTTTAAATTTA

CTTCGATCCAAGGGTATTTGATGTACCAGGTTCTTTCGATTACCTCTCACTCAAAATGACATTCCACTCA

AAGTCAGCGCTGTTTGCCTCCTTCTCTGTCCACAGAAATATCGCCGTCTCTTTCGCCGCTGCGTCCGCTA

TCTCTTTCGCCACCGTTTGTAGCGTTACGTAGCGTCAATGTCCGCCTTCAGTTGCATTTTGTCAGCGGTT

TCGTGACGAAGCTCCAAGCGGTTTACGCCATCAATTAAACACAAAGTGCTGTGCCAAAACTCCTCTCGCT

TCTTATTTTTGTTTGTTTTTTGAGTGATTGGGGTGGTGATTGGTTTTGGGTGGGTAAGCAGGGGAAAGTG

TGAAAAATCCCGGCAATGGGCCAAGAGGATCAGGAGCTATTAATTCGCGGAGGCAGCAAACACCCATCTG

CCGAGCATCTGAACAATGTGAGTAGTACATGTGCATACATCTTAAGTTCACTTGATCTATAGGAACTGCG

ATTGCAACATCAAATTGTATGCGGCGTGAGAACTGCGACCCACAAAAATCCCAAACCGCAATTGCACAAA

CAAATAGTGACACGAAACAGATTATTCTGGTAGCTGTTCTCGCTATATAAGACAATTTTTGAGATCATAT

CATGATCAAGACATCTAAAGGCATTCATTTTCGACTATATTCTTTTTTACAAAAAATATAACAACCAGAT

ATTTTAAGCTGATCCTAGATGCACAAAAAATAAATAAAAGTATAAACCTACTTCGTAGGATACTTCGGGG

TACTTTTTGTTCGGGGTTAGATGAGCATAACGCTAGTAGTTGATATTTGAGATCCCCTATCATTGCAGGG

TGACAGCGGAGCGGCTTCGCAGAGCTGCATTAACCAGGGCTTCGGGCAGGCCAAAAACTACGGCACGCTC

CGGCCACCCAGTCCGCCGGAGGACTCCGGTTCAGGGAGCGGCCAACTAGCCGAGAACCTCACCTATGCCT

GGCACAATATGGACATCTTTGGGGCGGTCAATCAGCCGGGCTCCGGATGGCGGCAGCTGGTCAACCGGAC

ACGCGGACTATTCTGCAACGAGCGACACATACCGGCGCCCAGGAAACATTTGCTCAAGAACGGTGAGTTT

CTATTCGCAGTCGGCTGATCTGTGTGAAATCTTAATAAAGGGTCCAATTACCAATTTGAAACTCAGTTTG

CGGCGTGGCCTATCCGGGCGAACTTTTGGCCGTGATGGGCAGTTCCGGTGCCGGAAAGACGACCCTGCTG

AATGCCCTTGCCTTTCGATCGCCGCAGGGCATCCAAGTATCGCCATCCGGGATGCGACTGCTCAATGGCC

AACCTGTGGACGCCAAGGAGATGCAGGCCAGGTGCGCCTATGTCCAGCAGGATGACCTCTTTATCGGCTC

CCTAACGGCCAGGGAACACCTGATTTTCCAAGCCATGGTGCGGATGCCACGACATCTGACCTATCGGCAG

CGAGTGGCCCGCGTGGATCAGGTGATCCAGGAGCTTTCGCTCAGCAAATGTCAGCACACGATCATCGGTG

TGCCCGGCAGGGTGAAAGGTCTGTCCGGCGGAGAAAGGAAGCGTCTGGCATTCGCCTCCGAGGCTCTAAC

CGATCCGCCGCTTCTGATCTGCGATGAGCCCACCTCCGGACTGGACTCCTTTACCGCCCACAGCGTCGTC

CAGGTGCTGAAGAAGCTGTCGCAGAAGGGCAAGACCGTCATCCTGACCATTCATCAGCCGTCTTCCGAGC

TGTTTGAGCTCTTTGACAAGATCCTTCTGATGGCCGAGGGCAGGGTAGCTTTCTTGGGCACTCCCAGCGA

AGCCGTCGACTTCTTTTCCTAGTGAGTTCGATGTGTTTATTAAGGGTATCTAGTATTACATAACATCTCA

ACTCCTATCCAGCGTGGGTGCCCAGTGTCCTACCAACTACAATCCGGCGGACTTTTACGTACAGGTGTTG

GCCGTTGTGCCCGGACGGGAGATCGAGTCCCGTGATCGGATCGCCAAGATATGCGACAATTTTGCCATTA

GCAAAGTAGCCCGGGATATGGAGCAGTTGTTGGCCACCAAAAATCTGGAGAAGCCACTGGAGCAGCCGGA

GAATGGGTACACCTACAAGGCCACCTGGTTCATGCAGTTCCGGGCGGTCCTGTGGCGATCCTGGCTGTCG

GTGCTCAAGGAACCACTCCTCGTAAAAGTGCGACTTATTCAGACAACGGTGAGTGGTTCCAGTGGAAACA

AATGATATAACGCTTACAATTCTTGGAAACAAATTCGCTAGATTTTAGATAGAATTGCCTGATTCCACAC

CCTTCTTAGTTTTTTTCAATGAGATGTATAGTTTATAGTTTTGCAGAAGATAAATAAATTTCATTTAACT

CGCGAATATTAATGAGATGCGAGTAACATTTTAATTTGCAGATGGTTGCCATCTTGATTGGCCTCATCTT

TTTGGGCCAACAACTCACGCAAGTGGGTGTGATGAATATCAACGGAGCCATCTTCCTCTTCCTGACCAAC

ATGACCTTTCAAAACGTCTTTGCCACGATAAATGTAAGTCATGTTTAGAATACATTTGCATTTCAATAAT

TTACTAACTTTCTAATGAATCGATTCGATTTAGGTGTTCACCTCAGAGCTGCCAGTTTTTATGAGGGAGG

CCCGAAGTCGACTTTATCGCTGTGACACATACTTTCTGGGCAAAACGATTGCCGAATTGCCGCTTTTTCT

CACAGTGCCACTGGTCTTCACGGCGATTGCCTATCCGATGATCGGACTGCGGGCCGGAGTGCTGCACTTC

TTCAACTGCCTGGCGCTGGTCACTCTGGTGGCCAATGTGTCAACGTCCTTCGGATATCTAATATCCTGCG

CCAGCTCCTCGACCTCGATGGCGCTGTCTGTGGGTCCGCCGGTTATCATACCATTCCTGCTCTTTGGCGG

CTTCTTCTTGAACTCGGGCTCGGTGCCAGTATACCTCAAATGGTTGTCGTACCTCTCATGGTTCCGTTAC

GCCAACGAGGGTCTGCTGATTAACCAATGGGCGGACGTGGAGCCGGGCGAAATTAGCTGCACATCGTCGA

ACACCACGTGCCCCAGTTCGGGCAAGGTCATCCTGGAGACGCTTAACTTCTCCGCCGCCGATCTGCCGCT

GGACTACGTGGGTCTGGCCATTCTCATCGTGAGCTTCCGGGTGCTCGCATATCTGGCTCTAAGACTTCGG

GCCCGACGCAAGGAGTAGCCGACATATATCCGAAATAACTGCTTGTTTTTTTTTTTTACCATTATTACCA

TCGTGTTTACTGTTTATTGCCCCCTCAAAAAGCTAATGTAATTATATTTGTGCCAATAAAAACAAGATAT

GACCTATAGAATACAAGTATTTCCCCTTCGAACATCCCCACAAGTAGACTTTGGATTTGTCTTCTAACCA

AAAGACTTACACACCTGCATACCTTACATCAAAAACTCGTTTATCGCTACATAAAACACCGGGATATATT

TTTTATATACATACTTTTCAAATCGCGCGCCCTCTTCATAATTCACCTCCACCACACCACGTTTCGTAGT

TGCTCTTTCGCTGTCTCCCACCCGCTCTCCGCAACACATTCACCTTTTGTTCGACGACCTTGGAGCGACT

GTCGTTAGTTCCGCGCGATTCGGTTCGCTCAAATGGTTCCGAGTGGTTCATTTCGTCTCAATAGAAATTA

GTAATAAATATTTGTATGTACAATTTATTTGCTCCAATATATTTGTATATATTTCCCTCACAGCTATATT

TATTCTAATTTAATATTATGACTTTTTAAGGTAATTTTTTGTGACCTGTTCGGAGTGATTAGCGTTACAA

TTTGAACTGAAAGTGACATCCAGTGTTTGTTCCTTGTGTAGATGCATCTCAAAAAAATGGTGGGCATAAT

AGTGTTGTTTATATATATCAAAAATAACAACTATAATAATAAGAATACATTTAATTTAGAAAATGCTTGG

ATTTCACTGGAACTAGGGCGCGCCTCCGGAACATAATGGTGCAGGGCGCTGACTTCCGCGTTTCCAGACT

TTACGAAACACGGAAACCGAAGACCATTCATGTTGTTGCTCAGGTCGCAGACGTTTTGCAGCAGCAGTCG

CTTCACGTTCGCTCGCGTATCGGTGATTCATTCTGCTAACCAGTAAGGCAACCCCGCCAGCCTAGCCGGG

TCCTCAACGACAGGAGCACGATCATGCGCACCCGTGGCCAGGGCCGCAAGCTTGCATGCCTGCAGGTCGG

AGTACTGTCCTCCGAGCGGAGTACTGTCCTCCGAGCGGAGTACTGTCCTCCGAGCGGAGTACTGTCCTCC

GAGCGGAGTACTGTCCTCCGAGCGGAGACTCTAGCCCTAGGGCATGCCTGCAGGTCGGAGTACTGTCCTC

CGAGCGGAGTACTGTCCTCCGAGCGGAGTACTGTCCTCCGAGCGGAGTACTGTCCTCCGAGCGGAGTACT

GTCCTCCGAGCGGAGACTCTAGCGCTAGCGACGTCGAGCGCCGGAGTATAAATAGAGGCGCTTCGTCTAC

GGAGCGACAATTCAATTCAAACAAGCAAAGTGAACACGTCGCTAAGCGAAAGCTAAGCAAATAAACAAGC

GCAGCTGAACAAGCTAAACAATCTGCAGTAAAGTGCAAGTTAAAGTGAATCAATTAAAAGTAACCAGCAA

CCAAGTAAATCAACTGCAACTACTGAAATCTGCCAAGAAGTAATTATTGAATACAAGAAGAGAACTCTGA

ATAATCAAA

**ATGAATGGAGCTATAGGAGGTGACCTTTTGCTCAATTTTCCTGACATGTCGGTCCTAGAGCGCCAAAGGGCTCACCTCAAGTACCTCAATCCCACCTTTGATTCTCCTCTCGCCGGCTTCTTTGCCGATTCTTCAATGATTACCGGCGGCGAGATGGACAGCTATCTTTCGACTGCCGGTTTGAATCTTCCGATGATGTACGGTGAGACGACGGTGGAAGGTGATTCAAGACTCTCAATTTCGCCGGAAACGACGCTTGGGACTGGAAATTTCAAGAAACGGAAGTTTGATACAGAGACTAAGGATTGTAATGAGAAGAAGAAGAAGATGACGATGAACAGAGATGACCTAGTAGAAGAAGGAGAAGAAGAGAAGTCGAAAATAACAGAGCAAAACAATGGGAGCACAAAAAGCATCAAGAAGATGAAACACAAAGCCAAGAAAGAAGAGAACAATTTCTCTAATGATTCATCTAAAGTGACGAAGGAATTGGAGAAAACGGATTATATTCATGCTAGCGGAGGAGGTGGTGGAGGTGGAGGTGGAGGTACTAGTAAAGCGTTAACGGCCAGGCAACAAGAGGTGTTTGATCTCATCCGTGATCACATCAGCCAGACAGGTATGCCGCCGACGCGTGCGGAAATCGCGCAGCGTTTGGGGTTCCGTTCCCCAAACGCGGCTGAAGAACATCTGAAGGCGCTGGCACGCAAAGGCGTTATTGAAATTGTTTCCGGCGCATCACGCGGGATTCGTCTGTTGCAGGAAGAGGAAGAAGGGTTGCCGCTGGTAGGTCGTGTGGCTGCCGGTGAACCACTTCTGGCGCAACAGCATATTGAAGGTCATTATCAGGTCGATCCTTCCTTATTCAAGCCGAATGCTGATTTCCTGCTGCGCGTCAGCGGGATGTCGATGAAAGATATCGGCATTATGGATGGTGACTTGCTGGCAGTGCATAAAACTCAGGATGTACGTAACGGTCAGGTCGTTGTCGCACGTATTGATGACGAAGTTACCGTTAAGCGCCTGAAAAAACAGGGCAATAAAGTCGAACTGTTGCCAGAAAATAGCGAGTTTAAACCAATTGTCGTAGATCTTCGTCAGCAGAGCTTCACCATTGAAGGGCTGGCGGTTGGGGTTATTCGCAACGGCGACTGGCTGGGAAGCGGAGAGGGCAGAGGAAGTCTGCTAACATGCGGTGACGTCGAGGAGAATCCTGGACCT**

ATGGTGAGCAAGGGCGAGGAGGATAACATGGCCATCATCAAGGAGTTCATGCGCTTCAAGGTGCACATGGAGGGCTCCGTGAACGGCCACGAGTTCGAGATCGAGGGCGAGGGCGAGGGCCGCCCCTACGAGGGCACCCAGACCGCCAAGCTGAAGGTGACCAAGGGTGGCCCCCTGCCCTTCGCCTGGGACATCCTGTCCCCTCAGTTCATGTACGGCTCCAAGGCCTACGTGAAGCACCCCGCCGACATCCCCGACTACTTGAAGCTGTCCTTCCCCGAGGGCTTCAAGTGGGAGCGCGTGATGAACTTCGAGGACGGCGGCGTGGTGACCGTGACCCAGGACTCCTCCCTGCAGGACGGCGAGTTCATCTACAAGGTGAAGCTGCGCGGCACCAACTTCCCCTCCGACGGCCCCGTAATGCAGAAGAAGACCATGGGCTGGGAGGCCTCCTCCGAGCGGATGTACCCCGAGGACGGCGCCCTGAAGGGCGAGATCAAGCAGAGGCTGAAGCTGAAGGACGGCGGCCACTACGACGCTGAGGTCAAGACCACCTACAAGGCCAAGAAGCCCGTGCAGCTGCCCGGCGCCTACAACGTCAACATCAAGTTGGACATCACCTCCCACAACGAGGACTACACCATCGTGGAACAGTACGAACGCGCCGAGGGCCGCCACTCCACCGGCGGCATGGACGAGCTGTACAAGTGTGGAAGCGGAGAGGGCAGAGGAAGTCTGCTAACATGCGGTGACGTCGAGGAGAATCCTGGACCT

**ATGGAGTTCCAGTACCTGCCCGATACGGATGACCGTCACCGTATCGAAGAAAAGCGGAAGCGAACCTATGAAACCTTCAAGTCCATCATGAAAAAGTCCCCCTTCTCGGGCCCCACGGACCCGCGCCCCCCGCCCCGTCGTATTGCGGTTCCTTCGCGCAGCAGTGCCAGCGTCCCCAAACCCGCACCGCAGCCCTACCCGTTCACTTCCTCCCTTAGCACGATTAACTATGATGAGTTCCCCACGATGGTGTTCCCCAGTGGACAAATTTCCCAGGCATCGGCACTGGCTCCGGCCCCACCGCAAGTCCTCCCCCAGGCGCCCGCTCCGGCACCGGCTCCCGCAATGGTGAGTGCTCTGGCCCAGGCCCCCGCTCCAGTCCCCGTGCTGGCGCCTGGACCCCCACAGGCAGTTGCCCCTCCTGCTCCGAAACCAACGCAGGCGGGCGAAGGAACCCTGAGCGAGGCCCTCTTGCAGCTTCAGTTCGATGACGAAGACTTGGGAGCCCTGCTGGGTAACAGCACAGACCCTGCCGTATTCACCGATCTCGCATCCGTGGACAACAGCGAGTTTCAGCAGCTCTTGAATCAGGGAATCCCGGTCGCACCTCATACCACAGAGCCCATGCTGATGGAATACCCGGAGGCTATCACGCGACTGGTGACCGGCGCACAGCGACCACCCGATCCAGCCCCTGCCCCACTGGGTGCCCCGGGTTTGCCCAATGGCCTCCTCAGCGGCGATGAGGATTTCTCCAGCATCGCTGATATGGATTTCTCCGCTTTGCTGAGCCAGATAAGCTCCGCTAGCGGAGGAGGTGGTGGAGGTGGAGGTGGAGGTACTAGTTCTAGAATGAAGATGGACAAAAAGACTATAGTTTGGTTTAGAAGAGACCTAAGGATTGAGGATAATCCTGCATTAGCAGCAGCTGCTCACGAAGGATCTGTTTTTCCTGTCTTCATTTGGTGTCCTGAAGAAGAAGGACAGTTTTATCCTGGAAGAGCTTCAAGATGGTGGATGAAACAATCACTTGCTCACTTATCTCAATCCTTGAAGGCTCTTGGATCTGACCTCACTTTAATCAAAACCCACAACACGATTTCAGCGATCTTGGATTGTATCCGCGTTACCGGTGCTACAAAAGTCGTCTTTAACCACCTCTATGATCCTGTTTCGTTAGTTCGGGACCATACCGTAAAGGAGAAGCTGGTGGAACGTGGGATCTCTGTGCAAAGCTACAATGGAGATCTATTGTATGAACCGTGGGAGATATACTGCGAAAAGGGCAAACCTTTTACGAGTTTCAATTCTTACTGGAAGAAATGCTTAGATATGTCGATTGAATCCGTTATGCTTCCTCCTCCTTGGCGGTTGATGCCAATAACTGCAGCGGCTGAAGCGATTTGGGCGTGTTCGATTGAAGAACTAGGGCTGGAGAATGAGGCCGAGAAACCGAGCAATGCGTTGTTAACTAGAGCTTGGTCTCCAGGATGGAGCAATGCTGATAAGTTACTAAATGAGTTCATCGAGAAGCAGTTGATAGATTATGCAAAGAACAGCAAGAAAGTTGTTGGGAATTCTACTTCACTACTTTCTCCGTATCTCCATTTCGGGGAAATAAGCGTCAGACACGTTTTCCAGTGTGCCCGGATGAAACAAATTATATGGGCAAGAGATAAGAACAGTGAAGGAGAAGAAAGTGCAGATCTTTTTCTTAGGGGAATCGGTTTAAGAGAGTATTCTCGGTATATATGTTTCAACTTCCCGTTTACTCACGAGCAATCGTTGTTGAGTCATCTTCGGTTTTTCCCTTGGGATGCTGATGTTGATAAGTTCAAGGCCTGGAGACAAGGCAGGACCGGTTATCCGTTGGTGGATGCCGGAATGAGAGAGCTTTGGGCTACCGGATGGATGCATAACAGAATAAGAGTGATTGTTTCAAGCTTTGCTGTGAAGTTTCTTCTCCTTCCATGGAAATGGGGAATGAAGTATTTCTGGGATACACTTTTGGATGCTGATTTGGAATGTGACATCCTTGGCTGGCAGTATATCTCTGGGAGTATCCCCGATGGCCACGAGCTTGATCGCTTGGACAATCCCGCGTTACAAGGCGCCAAATATGACCCAGAAGGTGAGTACATAAGGCAATGGCTTCCCGAGCTTGCGAGATTGCCAACTGAATGGATCCATCATCCATGGGACGCTCCTTTAACCGTACTCAAAGCTTCTGGTGTGGAACTCGGAACAAACTATGCGAAACCCATTGTAGACATCGACACAGCTCGTGAGCTACTAGCTAAAGCTATTTCAAGAACCCGTGAAGCACAGATCATGATCGGAGCAGCACCTGATGAGATTGTAGCAGATAGCTTCGAGGCCTTAGGGGCTAATACCATTAAAGAACCTGGTCTTTGCCCATCTGTGTCTTCTAATGACCAACAAGTACCTTCGGCTGTTCGTTACAACGGGTCAAAGAGAGTGAAACCTGAGGAAGAAGAAGAGAGAGACATGAAGAAATCTAGGGGATTCGATGAAAGGGAGTTGTTTTCGACTGCTGAATCTTCTTCTTCTTCGAGTGTGTTTTTCGTTTCGCAGTCTTGCTCGTTGGCATCAGAAGGGAAGAATCTGGAAGGTATTCAAGATTCATCTGATCAGATTACTACAAGTTTGGGAAAAAATGGTTGCAAATGA**

AGGATCTTTGTGAAGGAACCTTACTTCTGTGGTGTGACATAATTGGACAAACTACCTACAGAGATTTAAAGCTCTAAGGTAAATATAAAATTTTTAAGTGTATAATGTGTTAAACTACTGATTCTAATTGTTTGTGTATTTTAGATTCCAACCTATGGAACTGATGAATGGGAGCAGTGGTGGAATGCCTTTAATGAGGAAAACCTGTTTTGCTCAGAAGAAATGCCATCTAGTGATGATGAGGCTACTGCTGACTCTCAACATTCTACTCCTCCAAAAAAGAAGAGAAAGGTAGAAGACCCCAAGGACTTTCCTTCAGAATTGCTAAGTTTTTTGAGTCATGCTGTGTTTAGTAATAGAACTCTTGCTTGCTTTGCTATTTACACCACAAAGGAAAAAGCTGCACTGCTATACAAGAAAATTATGGAAAAATATTTGATGTATAGTGCCTTGACTAGAGATCATAATCAGCCATACCACATTTGTAGAGGTTTTACTTGCTTTAAAAAACCTCCCACACCTCCCCCTGAACCTGAAACATAAAATGAATGCAATTGTTGTTGTTAACTTGTTTATTGCAGCTTATAATGGTTACAAATAAAGCAATAGCATCACAAATTTCACAAATAAAGCATTTTTTTCACTGCATTCTAGTTGTGGTTTGTCCAAACTCATCAATGTATCTTATCATGTCTGGATCGATCTGGCCGGCCGTTTAAACGAATTCTTGAAGACGAAAGGGCCTCGTGATACGCCTATTTTTATAGGTTAATGTCATGATAATAATGGTTTCTTA
