# Supplementary figures and images for: Optogenetic Control of Gene Expression in Drosophila
Source: PLoS One. 2015 Sep 18;10(9):e0138181. doi: 10.1371/journal.pone.0138181 (PMC4575133; doi:10.1371/journal.pone.0138181)

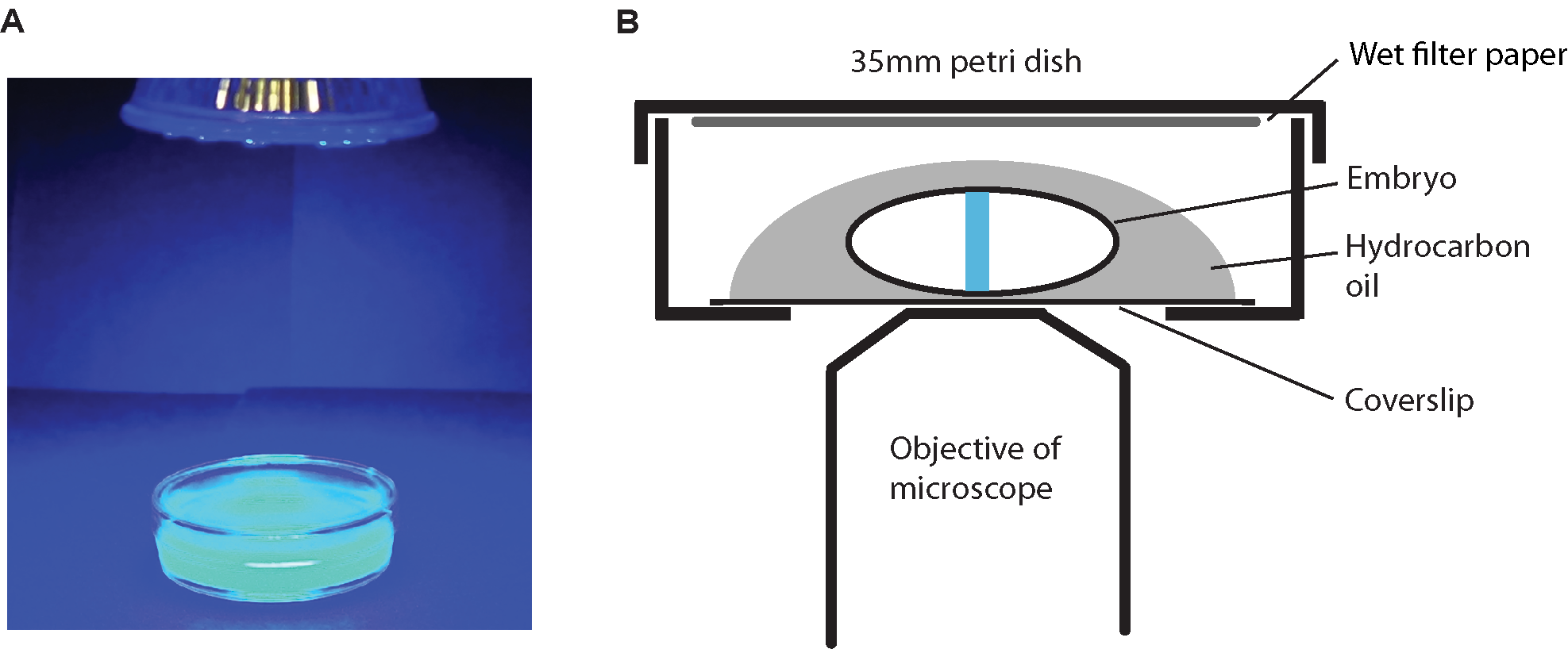

Supplement: S2 Fig — (A) Embryos were collected onto 1% agarose plates and illuminated from above using a commercial blue LED (474nm) (Superbrightleds MR16). (B) Schematic representation of the setup for two photon induction of embryo. Dechorionated embryos were aligned and glued onto coverslips. The coverslips were placed on top of a hole in a 35mm petri dish (MatTek). Wet filter paper and hydrocarbon oil was introduced to prevent embryos from desiccation during two photon induction. (TIF) [file pone.0138181.s002.tif]

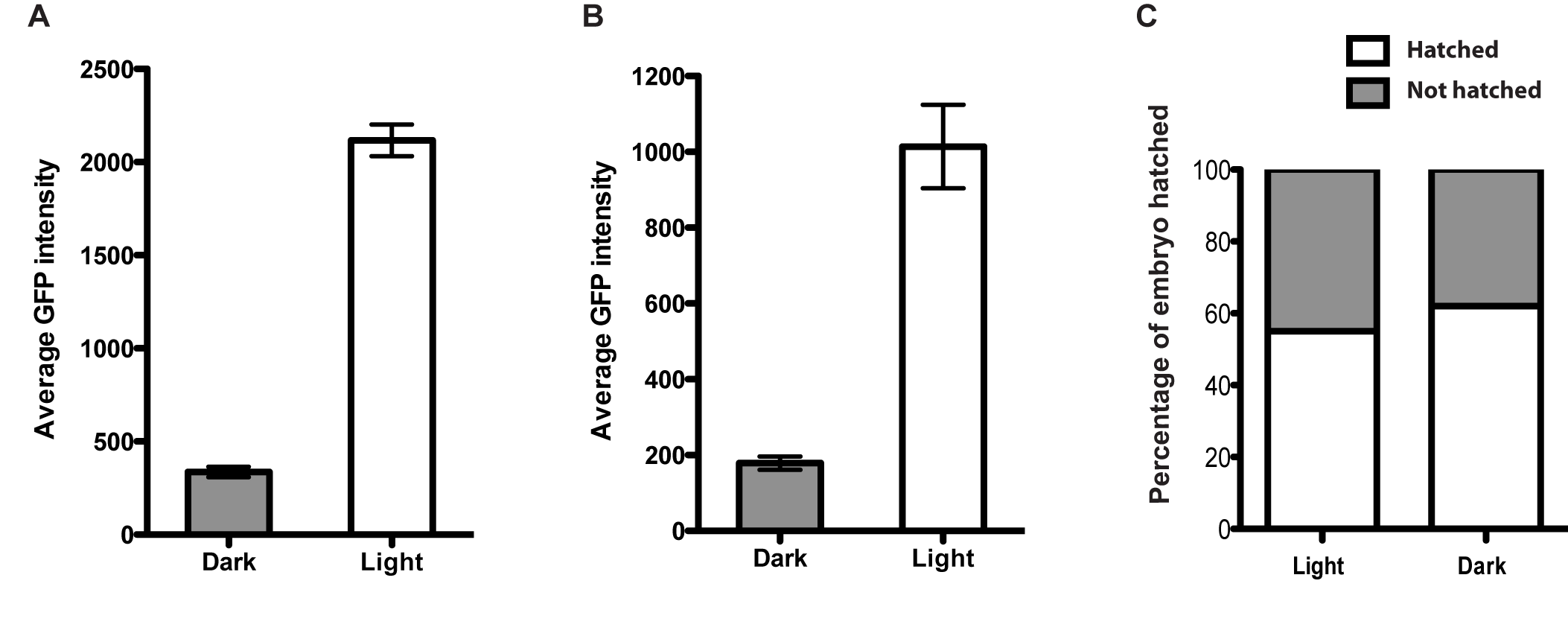

Supplement: S3 Fig — (A) Quantification of GFP expression in neuroblast cells in embryos. GMR18G07-Gal4 was used to drive the UAS- light inducible transcription system in neuroblast cells. Stage 15 embryos were illuminated with blue light (474nm, ~1.1mWcm-2) for 4 hours. Significant GFP expression was observed after light induction (n = 20, mean±s.e.m., t-test p<0.0001). (B) Quantification of GFP expression in olfactory neuron in adult flies after blue light induction. orco-Gal4 was used to drive the UAS- light inducible transcription system selectively in olfactory neurons. Adult flies were exposed to blue light (474nm, ~2.5mWcm-2) for 24 hours. Significant GFP expression was observed in the antennal lobes after light induction (n = 10, mean±s.e.m., t-test p<0.0001). (C) No significant effects were seen on survival rates of embryos. Embryos expressing the light inducible transcription system in the nervous system using a pan neuronal driver (elav c155-Gal4 UAS-CIBN::LexA-mcherry-p65::CRY2 LexAOP2-GFP LexAOP2-Gal80) were illuminated with blue light (474nm, ~2.5mWcm-2) throughout the entire period of embryogenesis. No lethality was observed comparing to the negative control incubated in dark (Fisher’s exact test, p>0.4). (TIF) [file pone.0138181.s003.tif]

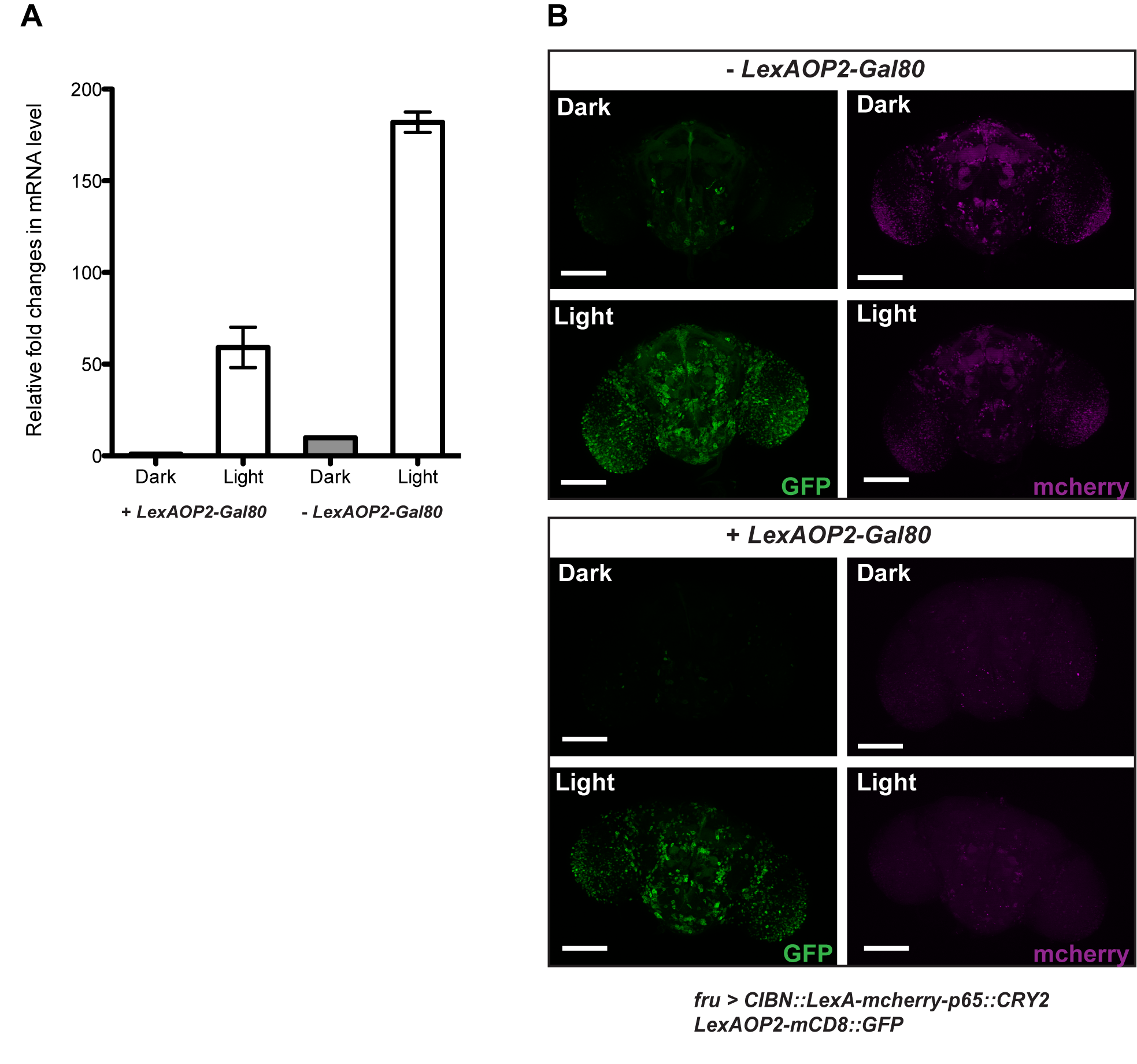

Supplement: S4 Fig — (A) Quantification of the GFP mRNA expression level using qPCR. Transgenic male flies (elav c155-Gal4 UAS-CIBN::LexA-mcherry-p65::CRY2 LexAOP2-GFP) with or without the LexAOP2-Gal80 feedback mechanism were either exposed to blue light (474nm, ~2.5mWcm-2) for ~18 hours or kept in constant darkness. Relative fold changes in mRNA amount were normalized against the un-induced flies with the LexAOP2-Gal80 feedback mechanism (n = 3, mean±s.e.m.). (B) Addition of a Gal80 feedback mechanism minimizes basal expression without illumination in fruitless neurons. Flies were illuminated with blue light for ~16 hours or kept in constant darkness. The incorporation of LexAOP2-Gal80 successfully lowered the basal GFP mRNA expression (Scale bar = 100μm). (TIF) [file pone.0138181.s004.tif]

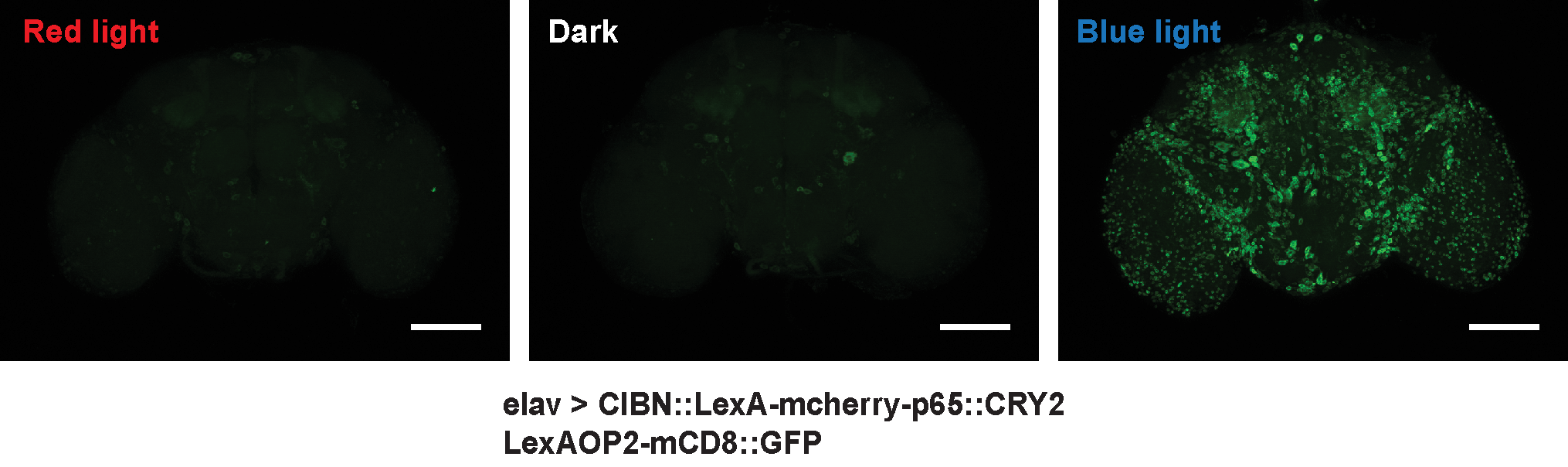

Supplement: S5 Fig — Induction of gene expression using the light switchable system depends on the wavelength of light. elav c155-Gal4 was used, including the LexAOP2-Gal80 feedback system, to drive the light inducible transcription system in adults. Flies were exposed to either red (626nm) or blue light (474nm, ~2.5mWcm-2) for 16 hours. Clear GFP expression was observed in adults illuminated with blue light only. Exposure to red light yielded little induction of GFP expression (Scale bar = 100μm). (TIF) [file pone.0138181.s005.tif]
